# Deoxynivalenol Biosynthesis in *Fusarium pseudograminearum* Significantly Repressed by a Megabirnavirus

**DOI:** 10.3390/toxins14070503

**Published:** 2022-07-19

**Authors:** Ke Li, Dongmei Liu, Xin Pan, Shuwei Yan, Jiaqing Song, Dongwei Liu, Zhifang Wang, Yuan Xie, Junli Dai, Jihong Liu, Honglian Li, Xiaoting Zhang, Fei Gao

**Affiliations:** 1 Department of Plant Protection, Henan Agricultural University, Zhengzhou 450002, China; mrleekk@126.com (K.L.); panxin241x@163.com (X.P.); yanshuwei0512@163.com (S.Y.); songjia-qing523@163.com (J.S.); ldw961225@163.com (D.L.); meretals@126.com (Z.W.); hnxieyuan@126.com (Y.X.); daijl666@126.com (J.D.); honglianli@sina.com (H.L.); 2 Institute of Agricultural Quality Standards and Testing Technology, Henan Academy of Agricultural Sciences, Zhengzhou 450002, China; dongmeiliu80@163.com (D.L.); ljha3100@163.com (J.L.)

**Keywords:** deoxynivalenol, *Fusarium pseudograminearum* megabirnavirus 1, mycoviruses, transcriptome

## Abstract

Deoxynivalenol (DON) is a mycotoxin widely detected in cereal products contaminated by *Fusarium.* Fusarium pseudograminearum megabirnavirus 1 (FpgMBV1) is a double-stranded RNA virus infecting *Fusarium pseudograminearum*. In this study, it was revealed that the amount of DON in *F. pseudograminearum* was significantly suppressed by FpgMBV1 through a high-performance liquid chromatography–tandem mass spectrometry (HPLC-MS/MS) assay. A total of 2564 differentially expressed genes were identified by comparative transcriptomic analysis between the FpgMBV1-containing *F. pseudograminearum* strain FC136-2A and the virus-free strain FC136-2A-V^-^. Among them, 1585 genes were up-regulated and 979 genes were down-regulated. Particularly, the expression of 12 genes (*FpTRI1*, *FpTRI3*, *FpTRI4*, *FpTRI5*, *FpTRI6*, *FpTRI8*, *FpTRI10*, *FpTRI11*, *FpTRI12*, *FpTRI14*, *FpTRI15,* and *FpTRI101*) in the trichothecene biosynthetic (*TRI*) gene cluster was significantly down-regulated. Specific metabolic and transport processes and pathways including amino acid and lipid metabolism, ergosterol metabolic and biosynthetic processes, carbohydrate metabolism, and biosynthesis were regulated. These results suggest an unrevealing mechanism underlying the repression of DON and *TRI* gene expression by the mycovirus FpgMBV1, which would provide new methods in the detoxification of DON and reducing the yield loss in wheat.

## 1. Introduction

*Fusarium* is a genus of filamentous fungi ubiquitously existing in agricultural and natural ecosystems [1,2]. Some species in *Fusarium* cause diseases including wilts, blights, rots, and cankers on crops and some horticultural, ornamental, and forest plants [3,4]. Wheat crown rot (FCR), one of the most destructive wheat diseases worldwide, is caused by species in *Fusarium* [5]. The dominant causal agent of FCR is *F. pseudograminearum* [6,7,8]. The symptoms of FCR include dry seedlings at the seedling stage, browning and rot at the base of the stem at the adult stage, withered white ears at the filling stage, and shriveled grains at the harvest stage [9]. Besides the significant decrease in wheat yield, *F. pseudograminearum* produces a diverse array of toxic secondary metabolites including mycotoxins [10,11]. Cereals contaminated with mycotoxins are unsuitable for food or feed [12,13]. The mycotoxins produced by *Fusarium* include trichothecenes, fumonisins, etc. The most common *Fusarium* mycotoxin is deoxynivalenol (DON), which causes the disease of emesis, oral lesions, dermatitis, and hemorrhaging in human and livestock [14,15]. The biosynthesis of DON starts from the cyclization of farnesyl pyrophosphate mediated by trichodiene synthases (encoded by *TRI5*) [16]. Then, multiple steps are followed by at least six additional enzymes encoded by *TRI4*, *TRI101*, *TRI11*, *TRI3*, *TRI1*, and *TRI8*. Moreover, two transcription factors, *TRI6* and *TRI10*; a transmembrane transporter, *TRI12;* and genes with unknown functions, *TRI9* and *TRI14,* may also be involved in DON biosynthesis and the virulence of *Fusarium* [17,18,19,20,21].

Mycoviruses are viruses hosted in fungi. They have been described in many fungal species. Some of the viruses are hypovirulence-related with an effect on reducing the virulence of the pathogenic fungi. Besides the hypovirulence effect, some mycoviruses have an influence on the cultivating features of host fungi including the morphology and development of the colonies and spores. For example, Cryphonectria hypovirus 1 (CHV1) decreased the pigmentation and spore production of *Cryphonectria parasitica* [22]. Sclerotinia sclerotiorum hypovirulence-associated DNA virus 1(SsHDV1) caused abnormal colony morphology and the reduction in colony growth and small sclerotia production to *Sclerotinia sclerotiorum* [23]. Rosellinia necatrix megabirnavirus 1 (RnMBV1) suppressed the growth and melanin biosynthesis in *Rosellinia* necatrix [24].

To date, 29 mycoviruses species have been reported in Fusarium spp. They belong to the families *Hypoviridae, Chrysoviridae, Totiviridae, Partitiviridae, Tymoviridae, Alternaviridae,* and *Megabirnaviridae* [25]. Among them, Fusarium graminearum virus 1 (FgV1) [26], Fusarium graminearum mycovirus-China 9 (FgV-ch9) [27], Fusarium graminearum hypovirus 2 (FgHV2) [28], and Fusarium pseudograminearum megabirnavirus 1 (FpgMBV1) were reported as hypovirulence-related viruses [29]. Hypovirulence-related viruses hosted in *F. graminearum*, specifically FgV1, FgV-ch9, and FgHV2, caused a significant reduction in the fungal vegetative growth [26,27,28]. FpgMBV1 hosted in *F. pseudograminearum* [29]. There were mild changes in the colony morphology and spore production, but significant reduction in the virulence of *F. pseudograminearum* to wheat caused by FpgMBV1 [30]. Moreover, there were two viruses in *F. graminearum*, FgV1 and Fusarium graminearum mycotymovirus 1 (FgMTV1/SX64), which were reported to have a reducing effect on the DON production. FgV1 had a hypovirulence effect on *F. graminearum*, while FgMTV1/SX64 had a mild effect on the virulence [26,31].

To reveal the mechanism underlying the regulation of mycoviruses on fungi, high-throughput mRNA sequencing (RNAseq) was applied. For instance, the differential expression of genes related to fungal metabolism, transcription, translation, and ribosomal RNA processing was shown to be related to FgV1 infection [32]. Host cell transport-related genes were down-regulated by Fusarium graminearum virus 3 (FgV3) [33]. Genes involved in RNA processing and ribosome transport assembly were down regulated during the infection of Fusarium graminearum virus 4 (FgV4) [33]. Genes related to glutamate metabolism, homoserine metabolism, cellular aldehyde metabolism, and lactate metabolism in *F*. *graminearum* were down-regulated by FgHV1 [34]. RNA silencing and virulence-related genes were considerably down-regulated in *S. sclerotiorum* strains infected by SsHADV-1 [35].

In this study, the DON production of the FpgMBV1-containing *F*. pseudograminearum strain was determined and compared with that of the virus-free strain by HPLC-MS/MS. The genome-wide transcriptional reprogramming in *F. pseudograminearum* under the infection of FpgMBV1 was outlined by RNA sequencing. Genes involved in the biosynthesis of DON and ergosterol and in RNA-silencing pathways were analyzed to unravel the interaction between FpgMBV1 and *F. pseudograminearum.* Data obtained in this study would provide a clue to the specific regulation mechanisms of mycovirus to DON biosynthesis and virulence of fungal pathogens including *Fusarium*.

## 2. Results

### 2.1. DON Synthesis Was Inhibited by Mycovirus FpgMBV1

**Figure 1:**
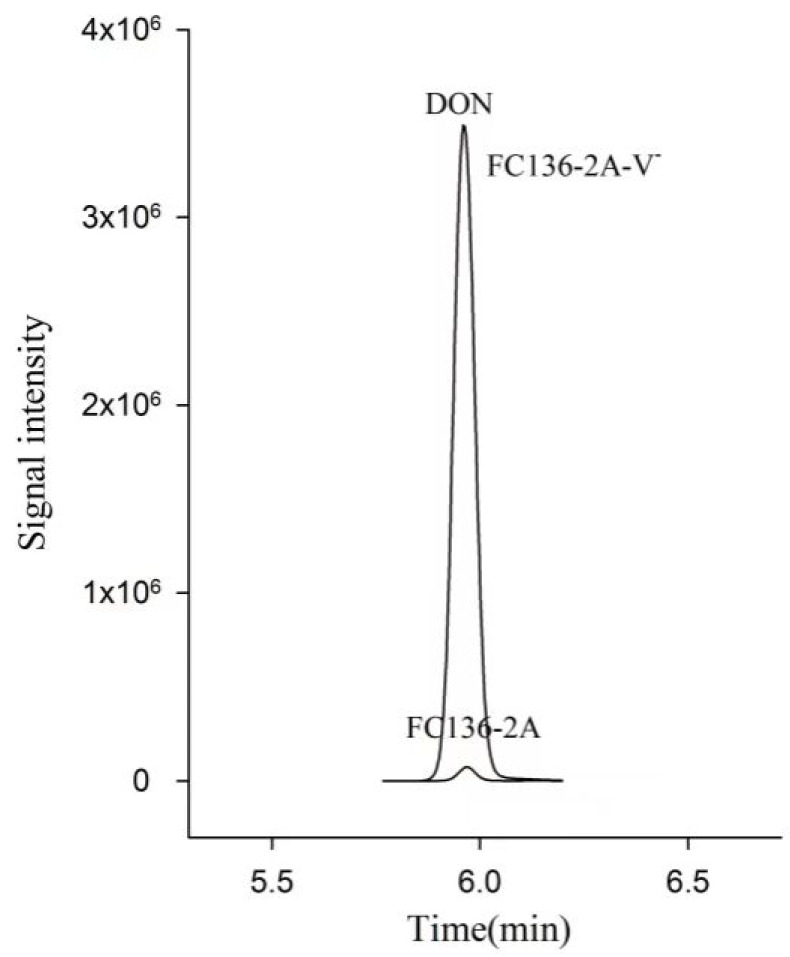
HPLC-MS/MS chromatograms showing DON production in wheat grain culture extracts of *F. pseudograminearum* strain FC136-2A harboring FpgMBV1 and the isogenic virus-free strain FC136-2A-V^-^.

To investigate the effect of FpgMBV1 on the content of DON in *F. pseudograminearum*, the isogenic strains FC136-2A and FC136-2A-V^-^ were cultivated in wheat grains for 30 days at 25 °C. Then, 20mL acetonitrile/water/acetic acid (70:29:1, *v*/*v*/*v*) was used to extract DON from 5 g of these mixtures for each sample for HPLC-MS/MS analysis. Results showed that the content of DON in the FpgMBV1-containing *F*. *pseudograminearum* strain FC136-2A was 1.3 ± 0.1 μg/kg. The content of DON in the virus-free strain FC136-2A-V^-^ was 10.6 ± 1.4 μg/kg. Compared to the virus-free strain FC136-2A-V^-^, the content of DON in the FpgMBV1-containing strain FC136-2A was reduced by about 87% (*p* < 0.05) (Figure 1). No significant difference was found for the content of 15-ADON in the FpgMBV1-containing strain and the virus-free strain (Figure S2).

### 2.2. Overview of RNA-Seq Data for FC136-2A and FC136-2A-V^-^

**Figure 2:**
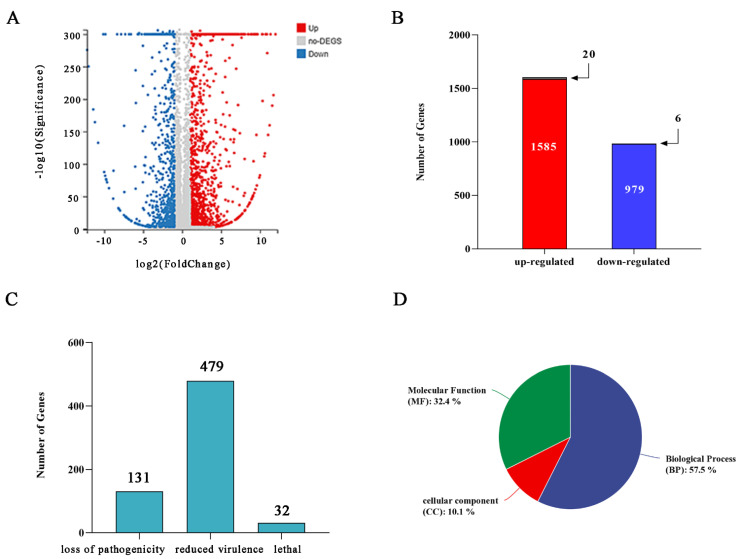
Effects of FpgMBV1 on the transcriptome profile of *F. pseudograminearum* as revealed by RNA-Seq. (**A**) The volcano plot showing gene signals detected in the strain FC136-2A comparing to FC136-2A-V^-^. Up-regulated (red), no change (gray) and down-regulated (blue). (**B**) The histogram of the number of differentially expressed genes (DEGs), including numbers of up-regulated (red) and down-regulated (blue) genes. (**C**) The number of DEGs annotated in the PHI database. (**D**) The rate of numbers of DEGs annotated by Gene ontology terms in molecular function (MF) (green), cellular component (CC) (red) and biological process (BP) (blue).

To reveal how the biosynthesis genes of DON were regulated by FpgMBV1, the FpgMBV1-containing *F. pseudograminearum* strain FC136-2A and the isogenic virus-free strain FC136-2A-V^-^ were used in the transcriptome analysis with three biological replicates for each strain using the DNBSEQ platform (BGI, Shenzhen, China). Gene expression profiles of the strains FC136-2A and FC136-2A-V^-^ were compared. An average of 7.24 Gb of data was yielded for each sample. The genome of *F. pseudograminearum CS3096* was used as the reference genome. An average of 94.57% and 94.27% of total reads (56–57 million) were aligned to the genome, respectively. A total of 11,500 genes were detected as expressed, of which 11417 were known and 83 were predicted as unknown genes (Table S1). The absolute fold change ≥ 2 and adjusted *p*-value ≤ 0.001 were used to define differently expressed genes (DEGs) (Figure 2A). Compared to the gene expression of the strain FC136-2A-V^-^, a total of 2564 statistically significant DEGs were found in the strain FC136-2A. Among them, 1585 genes were up-regulated and 979 genes were down-regulated (Figure 2B).

The PHI database annotation was screened using the DIAMOND software. A total of 131 genes were associated with loss of pathogenicity, 479 genes with reduced virulence, and 32 genes with lethal consequences (Figure 2C). In this study, the DEGs with PHI identity > 80% were used for further screening and the heat map construction. There were 18 genes categorized as reduced virulence genes including 4 up-regulated genes and 14 down-regulated ones (Figure S1). There were four genes categorized as essential genes (Figure S1). All these essential genes were up-regulated. No genes associated with loss of pathogenicity were identified in this study. Moreover, 12 of these genes were associated with DON and ergosterol biosynthesis (Table 1).

**Figure 3:**
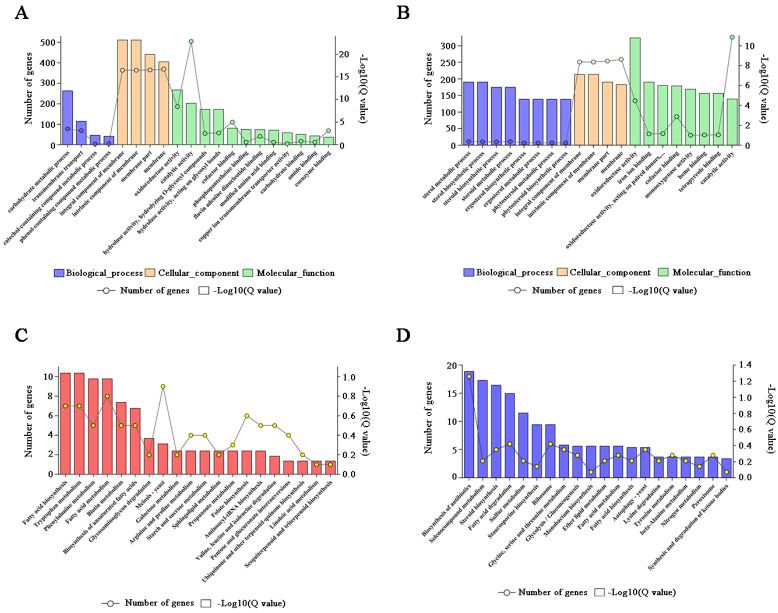
The Gene Ontology enrichment analysis of differently expressed genes, including up-regulated genes (**A**) and down-regulated genes (**B**) and the KEGG pathway enrichment analysis of the up-regulated genes (**C**) and down-regulated genes (**D**).

Gene ontology (GO) terms significantly enriched in the three major functional ontologies: 57.5% for biological process (BP), 10.1% for cellular component (CC) and 32.4% for molecular function (MF) (Figure 2D). For the 1585 up-regulated genes, the most enriched three for the BP were carbohydrate metabolic process, transmembrane transport, catechol-containing compound metabolic process, and oxidoreductase activity, catalytic activity, and cofactor binding for the MF (Figure 3A, Table S2). For the 979 down-regulated genes, the most enriched three of the BP were sterol metabolic and biosynthetic process, steroid metabolic and biosynthetic process, ergosterol metabolic and biosynthetic process; and oxidoreductase activity, iron ion binding, and cofactor binding for the MF (Figure 3B, Table S3). The most enrichedthree of the CC were integral component of membrane, an intrinsic component of membrane, membrane part, and membrane in all DEGs. There were 20 significantly enriched KEGG pathways for the up-regulated genes (Figure 3C, Table S4) and down-regulated genes, respectively (Figure 3D, Table S5). Sixteen pathways were related to metabolism.

### 2.3. The Metabolic Balance in F. pseudograminearum Was Disturbed by FpgMBV1

For the up-regulated DEGs, ten of the enriched KEGG pathways were related to metabolism, including tryptophan metabolism (ko00380), phenylalanine metabolism (ko00360), fatty acid metabolism (ko01212), biotin metabolism (ko00780), galactose metabolism (ko00052), arginine and proline metabolism (ko00330), starch and sucrose metabolism (ko00500), sphingolipid metabolism (ko00600), propanoate metabolism (ko00640), and linoleic acid metabolism (ko00591) (Figure 3C, Table S4). In addition, the meiosis–yeast pathway (ko04113) related to hyphal growth and development was also enriched.

Among the down-regulated genes, six pathways were related to metabolism (Figure 3D, Table S5). They were mainly classified into sulfur metabolism (ko00920), glycine, serine and threonine metabolism (ko00260), ether lipid metabolism (ko00565), fatty acid metabolism (ko01212), tyrosine metabolism (ko00350), and nitrogen metabolism (ko00910). Moreover, the biosynthesis of antibiotics, autophagy–yeast, steroid biosynthesis, and fatty acid degradation were also enriched.

**Figure 4:**
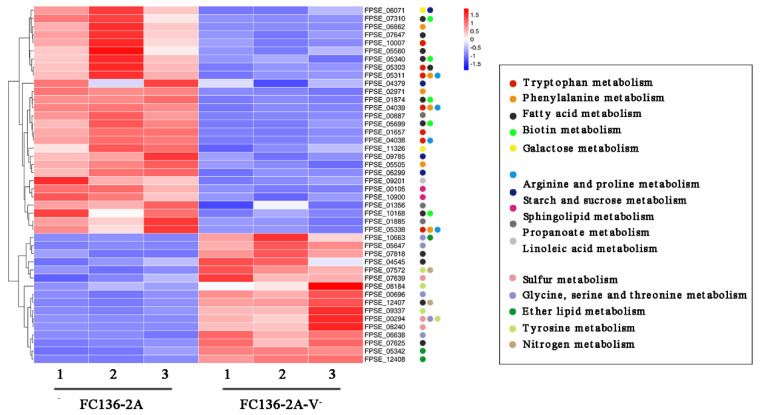
Heat map of metabolism-related genes differentially expressed in *F. pseudograminearum* strain FC136-2A compared to FC136-2A-V^-^.

The KEGG enrichment heat map shows that the fungal metabolic balance of amino acids and lipids was disturbed by FpgMBV1 (Figure 4). The transcription and translation of cell membrane-associated genes was also regulated by FpgMBV1.

### 2.4. TRI Genes Regulating DON Biosynthesis Were Down-Regulated by FpgMBV1

**Figure 5:**
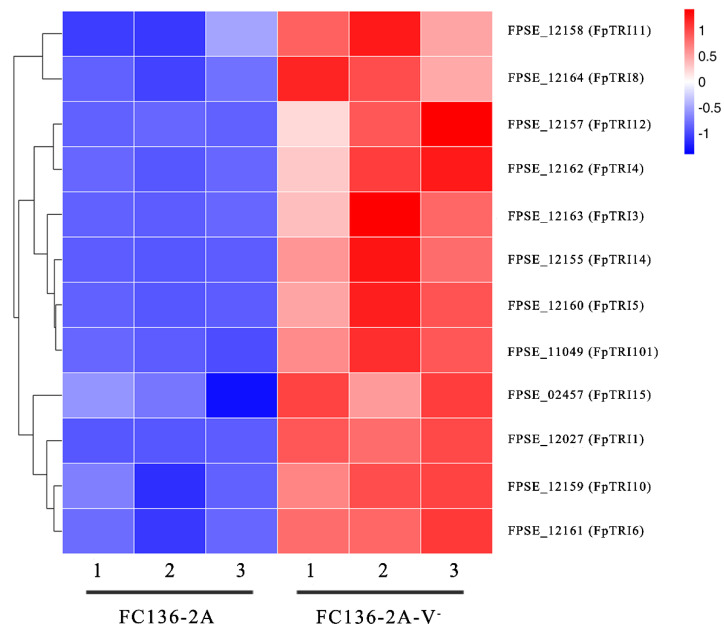
Heat map of TRI genes differentially expressed in F. pseudograminearum strain FC136-2A compared to FC136-2A-V^-^.

The genes in the TRI gene cluster encoding key enzymes for DON biosynthesis were FgTRI1-FgTRI16 and FgTRI101 in F. graminearum [36]. Comparative transcriptomic results showed that 12 genes in the TRI gene cluster were down-regulated by FpgMBV1. They were *FpTRI1*, *FpTRI3*, *FpTRI4*, *FpTRI5*, *FpTRI6*, *FpTRI8*, *FpTRI10*, *FpTRI11*, *FpTRI12*, *FpTRI14*, *FpTRI15,* and *FpTRI101* (Figure 5). These *TRI* genes regulated by FpgMBV1 accounted for about two-thirds of the total gene number in the TRI gene cluster. Additionally, they were all significantly repressed.

### 2.5. Ergosterol Biosynthesis and Metabolism Were Inhibited by FpgMBV1

**Figure 6:**
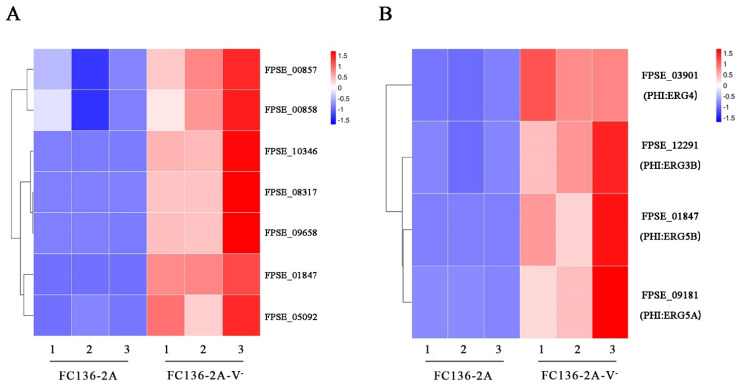
Heat map of genes related to ergosterol biosynthesis and metabolism differentially expressed in *F. pseudograminearum* strain FC136-2A compared to FC136-2A-V^-^, including genes annotated as ergosterol biosynthesis and metabolism based on NCBI-NR database (**A**), and based on PHI database (**B**).

Fungal ergosterol content is significantly and positively correlated with DON content. In this study, ergosterol synthesis and metabolism were significantly enriched according to the results of GO enrichment of down-regulated genes (Figure 6A). In the PHI database annotation, four DEGs related to ergosterol synthesis were significantly down-regulated by FpgMBV1. They were FgERG4 (PHI:2728), FgERG5B (PHI:3038), FgERG5A (PHI:3037), and FgERG3B (PHI:3036) (Figure 6B). These results showed inhibition in ergosterol biosynthesis and metabolism in the FpgMBV1-containing strain FC136-2A.

### 2.6. FpDicer1 and FpAGO1 Were Down-Regulated by FpgMBV1

**Figure 7:**
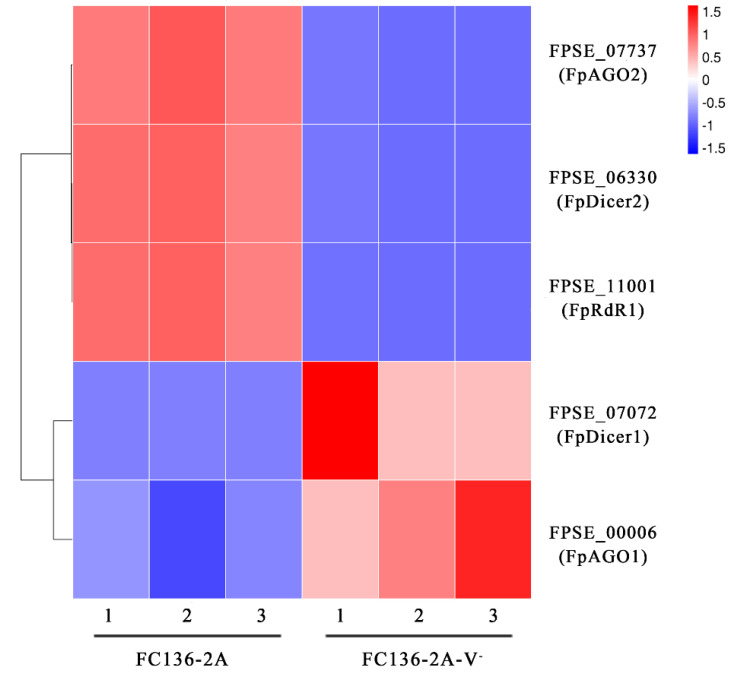
Heat map of RNA-silencing-related genes differentially expressed between *F. pseudograminearum* strain FC136-2A harboring FpgMBV1 and the isogenic virus-free strain FC136-2A-V^-^.

Based the RNA-seq result of FC136-2A and FC136-2A-V^-^, two Dicer (DCL) genes, two argonaute (AGO) genes, and one RNA-dependent RNA polymerase (RdR) gene were found differentially expressed. They were key genes in RNA silencing, which is an adaptive defense mechanism against foreign nucleic acids, especially viruses in animals, fungi, and plants [37]. Among them, FpDicer2(FPSE_06330), FpAGO2(FPSE_07737), and FpRdR1(FPSE_07737) were significantly up-regulated and FpDicer1(FPSE_07072) and FpAGO1(FPSE_00006) were down-regulated in the FpgMBV1-containing strain FC136-2A compared to the virus-free strain FC136-2A-V^-^ (Figure 7). These results suggest that these RNA-silencing-related genes in *F. pseudograminearum* were involved in the defensive immunity against FpgMBV1.

### 2.7. Gene Expression Level by Quantitive Real-Time RT-PCR

**Figure 8:**
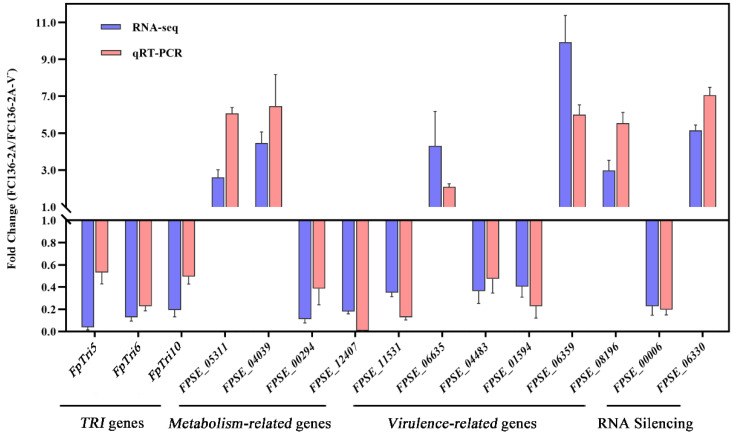
Gene expression comparison of some genes between *F. pseudograminearum* strain FC136-2A and FC136-2A-V^-^ by quantitative real-time RT-PCR (orange) and RNA-seq (blue).

Through quantitative real-time RT-PCR, the expression levels of 15 DEGs involved in metabolism, RNA silencing, virulence, and DON biosynthesis were confirmed. Primers are listed in Table S6. The expression levels of these representative genes were consistent with those in the transcriptomic data (Figure 8).

## 3. Discussion

In this study, the production of key mycotoxins DON and 15-ADON was found to be inhibited by FpgMBV1 significantly. The expression of 12 out of 15 *TRI* genes was repressed in accordance with the HPLC-MS/MS data. Previously, mycovirus FgV1 was reported to cause decreased DON production (60-fold) in *F. graminearum* [26]. However, only the *TRI12* gene in the TRI gene cluster was differently expressed in the FgV1-containing strains [32]. For FgMTV1/SX64, another mycovirus reducing the DON concentration significantly in *F. graminearum*, whether *TRI* gene expression was regulated is unknown [31]. The global regulation of most *TRI* genes by a mycovirus was first reported for FpgMBV1. Two transcription regulators, *TRI6* and *TRI10,* were key regulators in the *TRI* gene cluster [17]. *TRI6* has been identified as a global transcription regulator, not only enhancing the expression of the genes in the DON biosynthetic pathway but also involved in the upstream isoprenoid pathway for trichothecene accumulation [38,39]. *TRI10* regulated the expression of *TRI1, TRI3, TRI8, TRI11, TRI12, TRI14,* and *TRI15* in *Fusarium* spp. [40]. In this study, the significant down-regulation of *TRI6* and *TRI10* might be the reason for the down-regulation of other *TRI* genes. The significant disruption of *TRI* gene expression resulted in the low contents of DON mycotoxin. This suggests an unrevealing mechanism underlying the repression of DON and *TRI* gene expression by mycovirus FpgMBV1, which would provide new methods in the detoxification of DON and reducing the yield loss in wheat.

Other genes of biosynthetic pathways were differentially expressed under the infection of FpgMBV1. For example, four genes encoding key enzymes in ergosterol synthesis were down-regulated. They were *FgERG3B*, *FgERG4*, *FgERG5A*, and *FgERG5B* [41,42]. It has been reported that ergosterol synthesis has a strong, positive correlation with the content of mycotoxin DON in the infected grains [43,44]. Moreover, ergosterol is an important constituent of fungal membranes [45]. Membranes are essential for many cellular processes, including the defensing response against viruses in fungi [46,47]. Another significantly down-regulated gene, HMG-CoA-reductase (*HMR1*), is the key enzyme in the mevalonate pathway. *HMR1* is involved in the biosynthesis of many primary and secondary metabolites [48]. Some mevalonate derivatives function in fungi–plant interaction, such as isoprenoids and gibberellins [49]. Another down-regulated gene, *FgVELB,* plays a critical role in regulating various cellular processes and acts as a negative regulator for lipid biosynthesis. The deletion mutant of the *FgVELB* gene in *F. graminearum* produces a very low level of DON [50,51]. These results demonstrated the specific genes involved in the regulation of DON production Further studies on these genes would clarify the virulence-related pathways in fungi, especially *Fusarium*.

In general, the gene expression profile of *F. pseudograminearum* was reprogrammed by FpgMBV1 with 2564 genes differentially expressed. The disrupted metabolism caused by FpgMBV1 included amino acid and lipid metabolism, ergosterol metabolic and biosynthetic processes, carbohydrate metabolism and biosynthesis. Some of these pathways were critical in the transcriptome of other fungi under the infection of mycoviruses. In *C. parasitica*, “biosynthesis of other secondary metabolites”, “amino acid metabolism”, “carbohydrate metabolism”, and “translation” were enriched among the DEGs after CHV1 infection, demonstrating that virus infection resulted in massive but specific changes in primary and secondary metabolism. Some of the highly induced metabolites played key roles in the growth, development and pathogenicity of fungi [52]. The complex interaction between leucine metabolism and the global regulator of mycotoxin biosynthesis, *TRI6,* and virulence in *F. graminearum* has been explored [53]. In this study, the KEGG enrichment results showed that tryptophan, phenylalanine, tyrosine, glycine, serine, and threonine-related genes were differentially expressed under the infection of FpgMBV1. Further studies on the regulation mechanism of FpgMBV1 would help in revealing the crosstalk between some primary metabolic pathways and mycotoxin biosynthesis and virulence in *F. pseudograminearum*.

Mycoviruses were triggers and targets of RNA silencing. Three genes (*FpDCL2*, *FpAGO2*, and *FpRdR1)* were up-regulated and two genes (*FpDCL1* and *FpAGO1)* were down-regulated in *F. pseudograminearum* under the infection of FpgMBV1. They are genes for key components in the RNA-silencing machinery [54]. Gene expression of *DCL2* and *AGO2* were induced by CHV1 in *C. parasitica* [55]. Genes of *SsAgl2*, *SsDcl1*, and *SsDCl2* were essential in defending against viruses in *S. sclerotiorum* [56]. The expression of *FgDICER2* and *FgAGO2* were suppressed by FgV1 in *F. graminearum* [57]. Most RNA-silencing genes in *S. sclerotiorum* were repressed by SsHADV-1 [35]. Considering the high diversity of these virus–fungi systems, the different regulation on the RNA-silencing pathway seems reasonable. The regulation network of FpgMBV1 is partially revealed in this study, while the underlying mechanism is to be explored.

## 4. Conclusions

**Figure 9:**
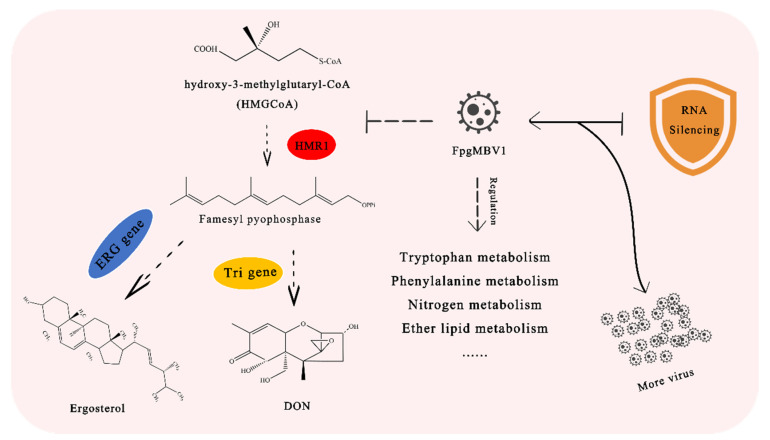
General view of genes and pathways regulated by FpgMBV1 in *F. pseudograminearum* revealed in this study.

Megabirnavirus FpgMBV1 significantly repressed the production of mycotoxin DON and the expression of 12 *TRI* genes in *F. pseudograminearum*. The expression of ergosterol biosynthesis and RNA-silencing-related genes and genes involved in metabolism were regulated by FpgMBV1(Figure 9). Prospectively, FpgMBV1 is valuable in the detoxification of DON and the management of diseases caused by *F. pseudograminearum*.

## 5. Materials and Methods

### 5.1. Fungal Material and Growth Conditions

FpgMBV1-containing *F*. *pseudograminearum* strain FC136-2A and its isogenic FpgMBV1-free strain FC136-2A-V^-^ were maintained in the laboratory, department of plant protection, Henan Agricultural University, Henan province, China [27]. For RNA sequencing, these two strains were grown at 25 °C in the dark on potato dextrose agar (PDA) medium (Becton, Dickinson, and Company, Sparks, MD, USA). For HPLC-MS/MS analysis, these strains were cultured in wheat grain media. Typically, 180 g of wheat seeds was soaked in distilled water for 12 h and boiled for 30 min then air-dried and autoclaved at 120 °C for 30 min in triangle flasks to complete the preparation of the wheat grain medium. Three-day-old PDA plugs of FC136-2A or FC136-2A-V^-^ mycelia were cultured in wheat grain media for 30 days in the dark at 25 °C.

### 5.2. HPLC-MS/MS Analysis of Type B Trichothecene

Finely ground wheat grain (5.00 g) was weighted and extracted with 20 mL acetonitrile/water/acetic acid (70:29:1, *v*/*v*/*v*). The suspension was blended for 5 min at 2500 rpm using multi-tube vortexer UMV-2. The homogenized solution was centrifuged for 10 min after cooling (T = 4 ℃, 8000 rpm). Then, 750 µL of the upper layer and 2 mL water were mixed in a 15 mL tube at 11,000 rpm for 10 min. The upper layer was cleaned via nylon filters and used for HPLC-MS/MS analysis.

HPLC analysis was performed using LC-MS-8060NX (Shimadzu, Japan) in gradient conditions. The separation of the toxin was performed using 100 nm × 2.1 mm, 1.9 µm (Thermo Fisher Scientific, Shanghai, China). The column temperature was set at 40 ℃ and the injection volume was 1 µL. Solvent A was 1 mmoL·L-1 ammonium formate in 0.1% formic acid and solvent B was methanol and 1 mmoL·L-1 ammonium formate in 0.1% formic acid. A gradient at a flow rate of 0.2 mL·min-1 was performed within 26 min (Table S7).

MS/MS was performed on an 8060NX triple quadrupole mass spectrometer equipped with a Turbo Ion-Spray electrospray ionization (ESI) source (Shimadzu, Japan) heated at 400 °C in the negative (DON, 3-ADON, and 15-ADON) and positive (ZEN, D3G, and NIV) ionization mode. Quantitation was performed using multiple-reaction monitoring (MRM) with a dwell time of 100 ms. The following transition reactions of DON, 3ADON,15ADON, ZEN, D3G, NIV, and the IS with the respective declustering potential (DP), collision energy (CE), and cell exit potential (CXP) in parentheses were recorded using the first mass transition for quantitation. DON: *m*/*z* 297.1 (DP −21.0 V, CE −11.0 V, CXP −25.0V), 3ADON: *m*/*z* 339.2 (DP −30V, CE −14V, CXP −25V), 15ADON: *m*/*z* 356.2 (DP −18V, CE −9.0V, CXP −23V), D3G: *m*/*z* 503.0 (DP +26V, CE +20V, CXP +29V), ZEN: *m*/*z* 317.2 (DP +16V, CE +24V, CXP +29V), and NIV: *m*/*z* 357.2 (DP +13V, CE +13V, CXP +28V).

Data are reported as mean values ± SD of three biological replications. Type B trichothecene (NIV, DON, FX, 3-ADON, and 15-ADON) yields are expressed as μg·kg -1 of dry fungal biomass. Values were compared at the 1% significance level using DPS software (control vs. treated).

### 5.3. Total RNA Extraction

The 0.5 mg mycelia of strains FC136-2A and FC136-2A-V^-^ were harvested from PDA plates and ground in liquid nitrogen. Total RNA isolation was conducted using TRIzol (Invitrogen, Carlsbad, CA, USA). The extracted RNA was treated with RNase-free DNase I (Promega, Madison, WI, United Kingdom). The concentration of the extracted RNAs was determined with Nanodrop system (NanoDrop, Madison, WI, USA). The RNA integrity was examined by the RNA integrity number (RIN) using an Agilent 2100 bioanalyzer (Agilent, Santa Clara, CA, USA).

### 5.4. cDNA Library Preparation and Sequencing

MGI Easy RNA Library Preparation Kit (BGI-Shenzhen, China) and DNA Clean Beads MGI Easy DNA Cleanbeads Kit (BGI-Shenzhen, China) were used to purify mRNA and synthesize cDNA and PCR amplification; then, purification of the PCR product was carried out following the kit instructions. The product was validated on the Agilent Technologies 2100 bioanalyzer for quality control. The double-stranded PCR products from the previous step were heated, denatured, and circularized by splint oligo sequencing to obtain the final library. The single-stranded circular DNA was formatted as the last library and amplified with phi29 polymerase to make a DNA nanoball, with more than 300 copies per molecule. DNBs were loaded into the patterned nanoarray, and 100 paired base reads were generated on the BGI seq500 platform (BGI-Shenzhen, China).

### 5.5. RNA-Seq Data Analysis

The raw data were filtered with BGI’s filtering software SOAPnuke (version: v1.4.0) to remove reads containing splices (splice contamination), reads with unknown base N content greater than 10%, and reads in which bases with a quality value of lower than 15 accounted for more than 20% of the total number of bases. Using HISAT2 software (version: v2.1.0; http://www.ccb.jhu.edu/software/hisat), the clean reads were mapped to the *F. pseudograminearum* CS3096 genome (Taxonomy ID: 1028729). Bowtie2 (version: v2.2.5; http://bowtie-bio.sourceforge.net/Bowtie2/index.shtml) was used to compare the reference gene sequences and RSEM (version: v1.2.8) was applied to calculate the expression levels of genes and transcripts. Pearson correlation coefficients between every two samples were obtained using the core function in the R software, and principal component analysis (PCA) was performed using the princomp method. To identify significant virulence genes in fungus, DIAMOND software was used to annotate genes with query coverage of 50% and identity of 40% in the PHI database (version: V0.8.31). To improve the accuracy of DEGs, we defined genes with more than two-fold difference and Q-values ≤ 0.001 to be screened as significantly differentially expressed genes. Differential genes were functionally classified according to GO and KEGG annotation results and official classification. At the same time, enrichment analysis was performed using the phyper function in R software, with FDR correction for *p*-value, and functions with Q value ≤ 0.05 were considered significantly enriched.

### 5.6. Quantitative Real-Time RT-PCR (qRT-PCR) Analysis

Total RNA samples from FC136-2A and FC136-2A-V^-^ were used for cDNA synthesis by Goldenstar^®^ RT6 cDNA Synthesis kit ver.2 (Tsingke Biotechnology Co., Ltd., Beijing, China). The resulting cDNAs were used as templates for qRT-PCR.

The qRT-PCR was carried out in a QuantStudio 3 Real-Time PCR System (Thermo Fisher Scientific, Waltham, MA, USA) with 2×T5 Fast qPCR Mix (Tsingke Biotechnology Co., Ltd., Beijing, China). PCR amplification was performed under the following conditions: 95 °C for 1 min, followed by 40 cycles of 95 °C for 10 s, and 60 °C for 30 s. Meltcurve plots were analyzed for each gene tested after each PCR reaction. The ubiquitin gene of TEF1α (FPSE_11980) served as an internal reference gene. Primers for 15 of the DEGs were designed using Primer Premier 5 and are listed in Table S6.

## Figures and Tables

**Table 1:** DON and ergosterol biosynthesis and metabolism-related genes according to the PHI database.

Gene Name	Gene ID	SwissProt_Description	log2(FC136_2A/FC136_2A_V^-^)	Identity (%)	E-Value	PHI Accession
*ERG3B*	*FPSE_12291*	Probable Delta(7)-sterol 5(6)-desaturase	−1.656	92.3	7.3 × 10^−179^	PHI:3036
*ERG4*	*FPSE_03901*	Delta(24(24(1)))-sterol reductase	−1.336	96.6	0	PHI:2728
*ERG5A*	*FPSE_09181*	Cytochrome P450 61	−1.584	99.3	0	PHI:3037
*ERG5B*	*FPSE_01847*	Sterol 22-desaturase	−7.320	94.3	1.6 × 10^−292^	PHI:3038
*TRI5*	*FPSE_12160*	Trichodiene synthase	−3.030	100	1.1 × 10^−228^	PHI:6846
*TRI6*	*FPSE_12161*	Trichothecene biosynthesis transcription regulator 6	−2.686	95.9	1.2 × 10^−126^	PHI:1362
*TRI12*	*FPSE_12157*	Trichothecene efflux pump TRI12	−2.305	91.7	4.5 × 10^−307^	PHI:2704
*TRI10*	*FPSE_12159*	Trichothecene biosynthesis transcription regulator 10	−2.702	93.3	1.5 × 10^−232^	PHI:2328
*TRI15*	*FPSE_02457*	Cys(2)-His(2) zinc finger protein	−1.803	95.7	8.3 × 10^−183^	PHI:1363
*HMR1*	*FPSE_03466*	Hydroxymethylglutaryl CoA reductase gene	−1.781	94.7	0	PHI:1006
*VELB*	*FPSE_11531*	Velvet complex subunit B	−1.760	83.3	1.1 × 10^−159^	PHI:2427
*GLX*	*FPSE_04483*	WSC domain-containing protein ARB_07867	−1.238	87	0	PHI:5393

## Data Availability

The data presented in this study are available in Supplementary Materials Tables S1–S7 and Figures S1 and S2.

## Supplementary Materials

The following supporting information can be downloaded at: https://www.mdpi.com/article/10.3390/toxins14070503/s1, Figure S1: The virulence genes according to the PHI-base. (A) Heat map of reduced-virulence genes in DEGs. (B) Heat map of essential genes (according to PHI database) in DEGs, Figure S2: Accumulation of mycotoxin 15-ADON in wheat cultures of FC136-2A and FC136-2A-V^-^. Table S1: Statistics of the transcriptome-sequencing results, Table S2: The GO enrichment analysis of the up-regulated genes, Table S3: The GO enrichment analysis of the down-regulated genes, Table S4: The KEGG enrichment analysis of the up-regulated genes, Table S5: The KEGG enrichment analysis of the down-regulated genes, Table S6: qRT-PCR primers used in this study, Table S7: Gradient elution conditions of mobile phase.
