# Supplementary figures and images for: Deoxynivalenol Biosynthesis in Fusarium pseudograminearum Significantly Repressed by a Megabirnavirus
Source: Toxins (Basel). 2022 Jul 19;14(7):503. doi: 10.3390/toxins14070503 (PMC9324440; doi:10.3390/toxins14070503)

**A**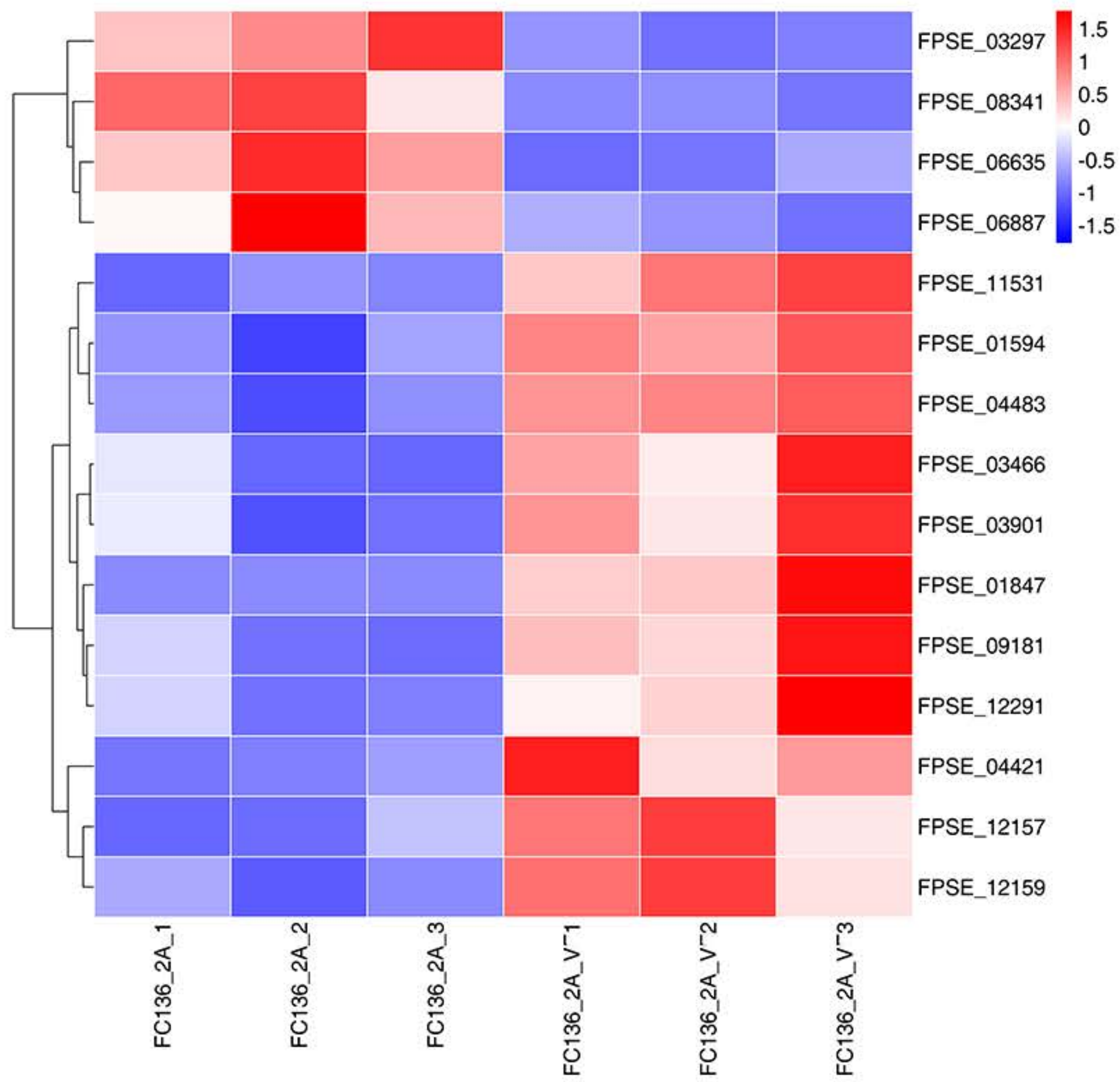**B**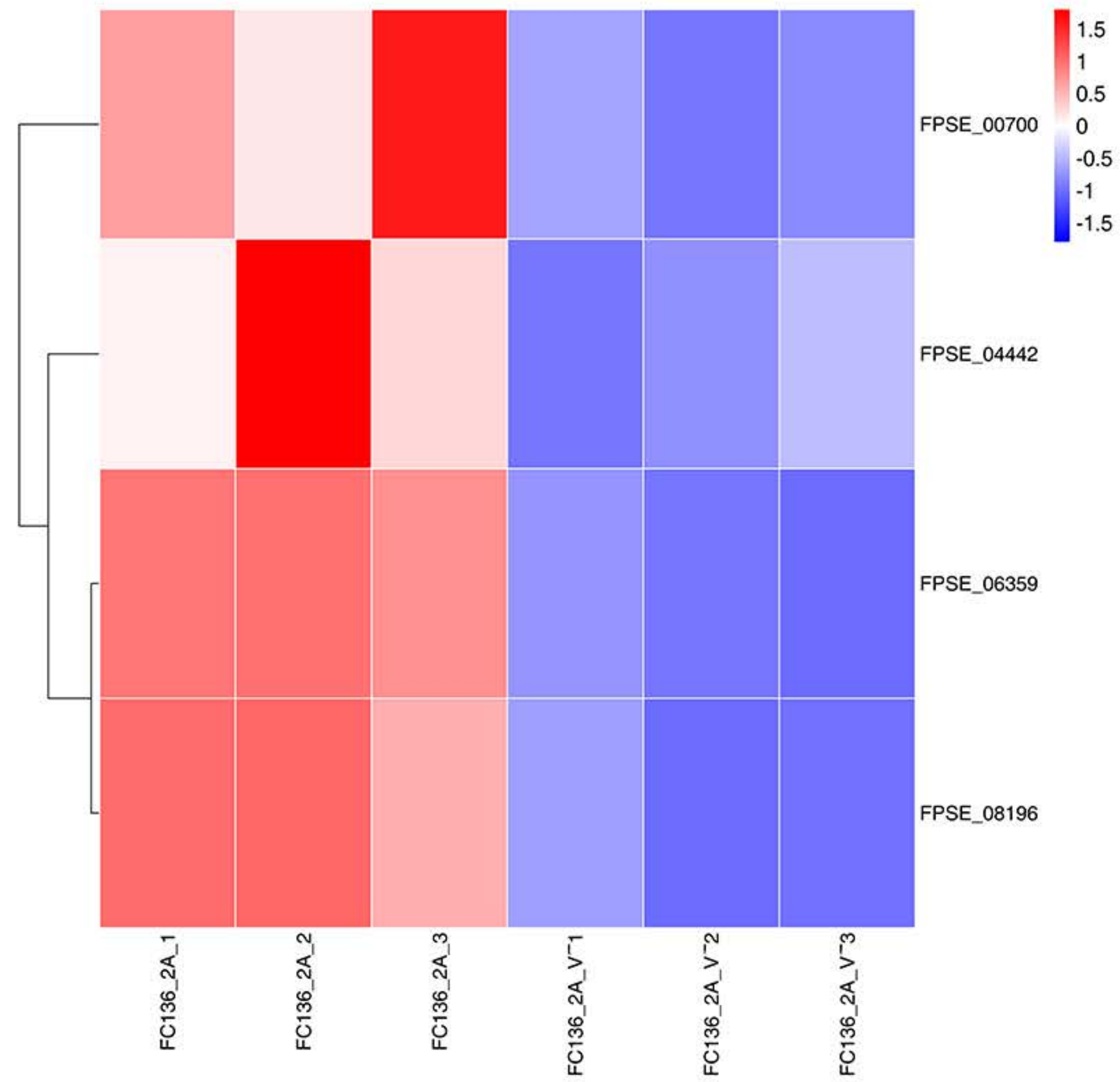

Supplement: Supplementary file 1 [file toxins-14-00503-s001.zip › Figure S1.pdf]

# FC136-2A

Q 356.20>321.20 (+)

5.33e3

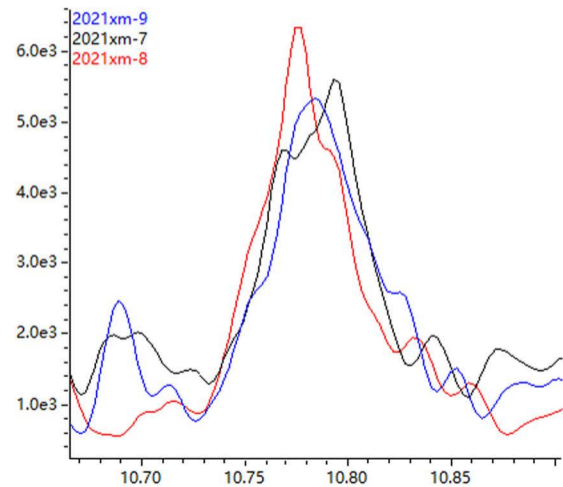

# FC136-2A-V-

Q 356.20>321.20 (+)

1.32e4

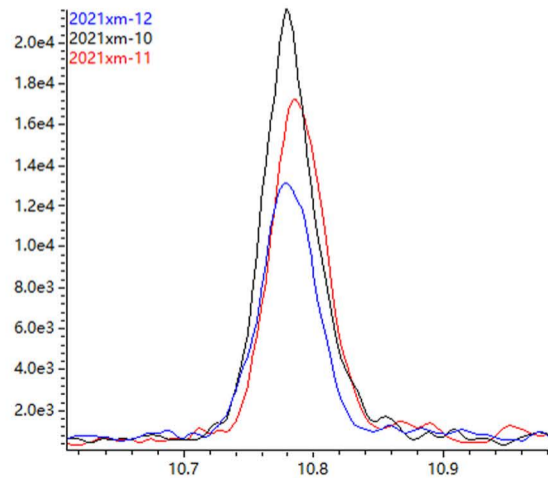

Supplement: Supplementary file 1 [file toxins-14-00503-s001.zip › Figure S2.pdf]
